# Quadriceps muscle power and optimal shortening velocity are inversely related to angiotensin converting enzyme activity in older men

**DOI:** 10.12688/f1000research.51208.2

**Published:** 2021-06-09

**Authors:** Joanna Kostka, Joanna Sikora, Agnieszka Guligowska, Tomasz Kostka

**Affiliations:** 1 Department of Gerontology, Medical University of Lodz, Lodz, Poland; 2 Laboratory of Bioanalysis, Department of Pharmaceutical Chemistry, Drug Analysis and Radiopharmacy, Medical University of Lodz, Lodz, Poland; 3 Department of Geriatrics, Healthy Ageing Research Centre, Medical University of Lodz, Lodz, Poland

**Keywords:** sarcopenia, muscle function, muscle strength, ageing, ACE inhibitors

## Abstract

**Background:** Methods which potentially could prevent age-related loss of muscle mass and function are still being sought. There are various attempts to use pharmacological agents to prevent loss of muscle mass, but the effectiveness of many of them still needs to be confirmed. One of the promising therapeutics are Angiotensin Converting Enzyme Inhibitors (ACEIs) and lowering of serum ACE activity. The goal of this study was to assess if taking Angiotensin Converting Enzyme Inhibitors (ACEI) and other angiotensin system blocking medications (ASBMs) can modify muscle performance in older men as well as to assess the association of serum ACE activity with muscle strength, power, muscle contraction velocity and functional performance.

**Methods:** Seventy-nine older men took part in the study. Muscle function was assessed with hand grip strength, maximum power relative to body mass (Pmax) and optimal shortening velocity (Ʋopt) of the knee extensor muscles. Anthropometric data, ACE activity and functional performance were also measured.

**Results:** Negative correlations between ACE activity and Pmax (rho=-0.29, p=0.04) as well as Ʋopt  (rho=-0.31, p=0.03) in a group of patients not taking ACEI and between ACE activity and Ʋopt (rho=-0.22, p=0.05) in the whole group of men were found. Positive relationship between age and ACE activity was demonstrated (rho=0.26, p=0.02). Age was the only selected variable in the multiple regression analyses to determine both Pmax and Ʋopt.

**Conclusions:** Serum ACE activity negatively associates to muscle power and muscle contraction velocity. The issues related to the impact of taking ACEI on the maintenance of muscle function and functional performance in older man require further studies.

## Introduction

Age-related sarcopenia, connected with deteriorating in muscle mass and function, is one of the most important factors determining functional status ^1– 3^. That is why methods used to prevent age-related loss of muscle mass and function are still being sought. Physical activity especially with proper nutritional intervention may be potentially an effective management for sarcopenia ^4^, but it is difficult to convince seniors to follow these recommendations. There are various attempts to use pharmacological agents to prevent loss of muscle mass, but the effectiveness of many of them still needs to be confirmed ^5^. Taking Angiotensin Converting Enzyme Inhibitors (ACEI) is also being considered. ACEI are traditionally used among others as anti-hypertensive medications and in cardiovascular diseases or diabetic nephropathy treatment ^6^, but there are also some hypotheses that using ACEI may modify muscle aging. For example, Onder *et al.* ^7^ in a three-year observational study in a group of older women with hypertension and without congestive heart failure have shown that ACEI treatment may slow age-related decline in muscle strength. The authors of the article explain this effect with several mechanisms, such as: direct influence on mechanical and metabolic changes in muscles (shift of the myosin heavy chains of skeletal muscle toward the fatigue-resistant forms; increasing insulin sensitivity, glycogen storage and glucose uptake in skeletal muscle; reduction of kinin breakdown and, consequently, improved circulation and better glucose and amino acid uptake), suppression of inflammatory activation connected with muscle catabobolism, beneficial effect on the nutritional status (inhibition of interleukin-6, which, among others, reduces appetite and, consequently, may cause malnutrition) ^7^.

Taking ACEI as well as the ACE genotype with insertion (II) of allele in gene, is associated with lower serum ACE activity ^8, 9^. There are suggestions that the ACE genotype type predisposes one to be successful in certain sports ^10^. ACE is a basic component of renin-angiotensin system (RAS), which converts angiotensin I (ang I) in angiotensin II (ang II). Ang II may in turn influence muscle performance ^11^. Williams *et al.* ^12^ reported that circulating ACE activity was correlated with isometric and isokinetic strength of quadriceps muscle strength in untrained man.

Even more important than muscle strength factor affecting functional performance in elderly population are muscle power and muscle contraction velocity ^1, 2^. These factors are also more influenced by aging than muscle strength ^13^. In addition to our recent research ^14^, there are no reports on the effect of ACEI taking and serum ACE activity on muscle power and muscle contraction velocity in older population. In that study we have found association between serum ACE activity with optimal shortening velocity of quadriceps muscles in older women, but not with muscle strength and power. Because various sex-related factors can modify age-related muscle changes ^15^, earlier reported sex-related differences in ACE activity ^16^, as well as possible differences in absorption of medications (absorption in women may be slower due to reduced gastric acid secretion and gastrointestinal motility) ^17^ we conducted similar study in the group of older men.

The goal of our study was to assess if taking ACEI can modify muscle performance in older man as well as to assess the association of serum ACE activity with muscle strength, power, muscle contraction velocity and functional performance

## Methods

### Subjects

Seventy-nine community-dwelling older men took part in the study. Participants were recruited through the local media by the Medical University of Lodz, Department of Geriatrics. The study included men aged ≥ 60 years, with the inclusion criteria of those with the ability to understand and execute commands, the ability to perform exercise testing, and those who signed the informed consent to participate in the study. Exclusion criteria were as follows: recent (<three months) diagnosis of myocardial infarction, stroke or orthopaedic surgery, cardiac contraindications to exercise tests, or lack of ability to perform tests because of motor system dysfunctions (limited range of motion, pain).

### Ethical statement

The study was approved by the Bioethics Committee of the Medical University of Lodz No RNN/647/14/KB.

### Protocol

Participants were asked to report to the Research Center in the morning (8–9 a.m.) on an empty stomach (at least 12 hours fasting), after a night’s sleep. They were also asked for avoid smoking, drinking alcohol and taking heavy physical exertion for at least 12 hours. After fasting blood drawing and a light breakfast, a comprehensive assessment was carried out with each participant. The assessment included: interview (information on socio-economic status, current and previous illnesses, and current medication), anthropometric measurements, evaluation of muscle function (muscle strength, power and shortening velocity), as well as functional status assessment.

Based on the analysis of medications taken, men were assigned to the following groups: ACEI - if they were taking any medications belonging to the ACEI group (n = 26) and to the non ACEI group - if they were not taking ACEI (n = 53). Similarly, if men were taking any drugs from the angiotensin system blocking medications (ASBMs) group, they were assigned to the ASBMs group (n = 34), if not, then to the non ASBMs group (n = 45)

### Anthropometric data

Measurements of height, weight, and skinfold thickness (from: triceps, biceps, sub-scapula, and supra-ileum) were performed using standard methods. Body mass index – BMI (kg∙m ^-2^) was calculated and the percentage of body fat was estimated according to Durnin & Womersley ^18^.

### Muscle strength

The Jamar ^®^ hydraulic hand dynamometer (Sammons Preston Rolyan, Bolingbrook, Canada) was used for handgrip strength evaluation. The test was preceded by a demonstration of the researcher and performed twice (on both sides), with 30 second pauses between measurements. During the test, the participant was in a standing position, with shoulder in neutral position and elbow in flexion (90°) with no radioulnar deviation, and was encouraged to squeeze the device as hard as possible. Results were given in kilograms (kg). A better record for each hand separately was used for analysis ^19^.

### Maximum power (Pmax) and optimal shortening velocity (Ʋopt) of the knee extensor muscles

Muscle power and optimal shortening velocity evaluations were performed with especially prepared ergometer (Monark type 818E Stockholm, Sweden), during two 8-second attempts to pedal at the maximum possible speed with friction loads of 0.25 N·kg ^−1^ and 0.35 N·kg ^−1^ of body mass. Instantaneous pedaling velocity (Ʋ), force (F) and power output (P) were calculated each 5ms and then averaged over each downstroke period. The highest value of P (maximal short-term power – Pmax) and optimal shortening velocity (Ʋopt - velocity at which the power reaches a maximum value) were calculated from a 3rd order polynomial function. The methodology used in this measurement has been previously described in detail ^20^. Pmax was presented in relation to body mass (W·kg ^-1^). Ʋopt was given in number of rotations per minute (rot·min ^-1^).

### Functional performance

Activities of Daily Living (ADL) ^21^, the Lawton Instrumental Activities of Daily Living (IADL) ^22^, and Timed Up & Go test (TUG) ^23^ were used to assess functional status of the participants.

ADL and IADL are scales based on questions concerning ability to perform basic and complex (instrumental) daily activities. ADL scale consists of six questions about activities such as eating, dressing, bathing, continence, toileting, and transferring. IADL contains questions about more complex activities enabling independent functioning, such as ability to use telephones, shopping, food preparation, cleaning, washing, use of public transport, responsibility for own medications, and ability to handle finances.

Timed Up & Go test is a simple and very popular functional test. It involves standing up from a sitting position, walking forth and back three meters, and sitting back on time. Time is given in seconds. After a familiarization test, this was performed twice and the better result was chosen for further analysis.

### ACE activity evaluation

Fasting blood samples were collected from every man at the Research Center in the morning hours into vacuum tubes. The blood was centrifuged and then stored at -20 °C until measurement. The reagents used and the method of analysis were described in our previous work ^14^. Results were given in U/L.

### Statistical analysis

The descriptive statistics are presented as mean ± standard deviation for data with a normal distribution, and for data without a normal distribution they were additionally shown as median (lower quartile - upper quartile). After the normality check, the one-way analysis of variance (ANOVA) or Mann-Whitney test (for quantitative data) and Chi ^2^ with Yates’ correction test (for qualitative data - prevalence of diseases) were used for group comparison. Spearman’s correlation coefficients were calculated to assess the relationships between numerical variables. To establish the most important determinants of Pmax and Ʋopt, the multiple regression analyses were made. For these analyses, ACE activity results were transformed logarithmically due to the non-normal distribution. Significance was set at a p value of p<0.05.

## Results

Baseline characteristics of the men taking and not taking ACEI as well as taking and not taking angiotensin system blocking medications (ASBMs) are shown in Table 1. Participants taking ACEI did not differ in terms of age, anthropometric indicators, education level, grip strength, muscle power, contraction velocity, and ADL results. Patients taking ACEI took more medications and were characterized by lower level of ACE in blood samples and lower IADL status. Men from both groups did not differ with regard to the prevalence of most diseases (ischemic heart disease, stroke, cancer, osteoporosis, COPD, heart failure, diabetes, myocardial infarction), but men talking ACEI more often suffered from hypertension (p=0.0003).

**Table 1.** Comparison of the results of the studied groups (ACEI vs non ACEI and ASBMs vs non ASBMs).

Characteristics	ACEI (n=26)	non ACEI (n=53)	ASBMs (n=34)	non ASBMs (n=45)
Age (years)	77.6±5.3 78.5 (75.0-82.0)	76.8±6.2 77.0 (72.0-80.0)	78.4±5.9	76.07±5.7
Body mass (kg)	82.3±13.4 82.0 (74.5-86,6)	79.6±10.9 78.0 (72.0-86.5)	82.71±13.4 81.8 (74.5-86.6)	78.8±10.1 78.0 (72.0-86.5)
Body mass index (kg·m ^-2^)	28.5±4.4 27.5 (26.2-29.2)	27.3±3.5 26.8 (24.5-28.7)	28.9±4.5 27.8 (26.2-30.9)	26.8±3.0 * 26.6 (24.4-28.1) *
Medications (number)	6.3±2.4	4.3±2.63 ^†^	6.5±2.2	3.9±2.5 ^#^
Fat percentage of body mass (%)	25.3±4.9	24.0±6.3	25.8±5.6	23.4±6.6
Left handgrip strength (kg)	35.3±7.8 35.0 (29.0-40.0)	38.8±10.21 36.0 (32.0-44.5)	34.6±8.6 34.0 (28.0-39.0)	39.9±9.7 * 37.5 (32.5-45.5) *
Right handgrip strength (kg)	37.8±9.2 36.5 (30.0-45.0)	39.8±10.6 37.5 (32.0-45.5)	37.1±9.1 35.5 (30.0-45.0)	40.8±10.6 38.5 (33.0-47.0)
Pmax (W·kg ^-1^)	3.89±1.45	4.31±1.48	3.84±1.40	4.42±1.49
Ʋopt (rep/min)	76.4±16.7 78.1 (66.7-85.7)	79.7±13.3 81.2 (70.6-89.4)	75.7±17.5	80.8±11.4
ACE activity (U L ^-1^)	40.0±22.2 39.3 (23.8-54.1)	55.0±30.0 * 46.1 (32.4-66.2) *	47.9±28.8 44.1 (26.0-56.9)	51.6±28.2 44.0 (31.3-65.6)
ADL	5.6±0.8 6.0 (5.5-6.0)	5.7±0.6 6.0 (6.0-6.0)	5.6±0.09 6.0 (5.5-6.0)	5.8±0.4 6.0 (6.0-6.0)
IADL	7.6±1.1 8.0 (8.0-8.0)	8.0±0.1 * 8.0 (8.0-8.0) ^†^	7.7±0.9 8.0 (8.0-8.0)	8.0±0.1 8.0 (8.0-8.0) *
TUG (s)	7.2±1.4 7.0 (6.0-8.0)	6.7±1.5 6.43 (5.7-7.4)	7.2±1.6 6.9 (6.0-8.0)	6.6±1.3 6.4 (5.7-7.4)

Note: The results for data with a normal distribution are presented as mean ±SD. The results for data without a normal distribution are presented as mean ± SD and additionally as median and quartiles *p<0.05; †p<0.01; #p<0.001 – ACEI compared to non ACEI and ASBMs compared to non ASBMs Abbreviations: Pmax, maximum power; Ʋopt, optimal shortening velocity of the knee extensor muscles; ACE, angiotensin-converting enzyme; ACEI, Angiotensin Converting Enzyme Inhibitors; ASBMs, angiotensin system blocking medications; ADL, Activities of Daily Living; IADL, Instrumental Activities of Daily Living; TUG, Timed Up and Go test; ACEI - men taking ACEI; non ACEI – men not taking ACEI; ASBMs - men taking ASBMs; non ASBMs - men not taking ASBMs

Whereas participants taking ASBMs were more than two years older than subjects not taking ASBMs, they took more medications, had greater BMI, and did not differ in terms of majority of muscles function parameters (Pmax, Ʋopt, right handgrip strength), except for the left handgrip strength.

**Figure 1.**
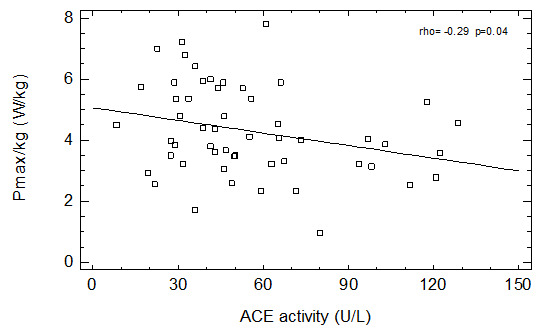
Correlation between ACE activity and Pmax in patients not taking ACEI.

**Figure 2.**
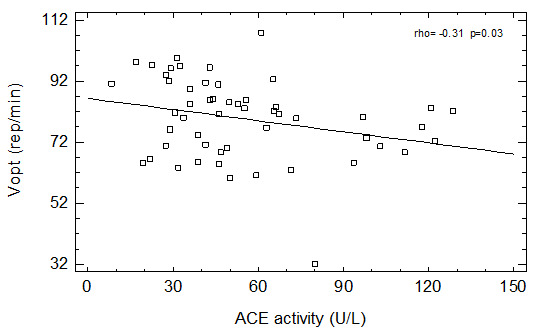
Correlation between ACE activity and Ʋopt in patients not taking ACEI.

We have found a positive relationship between age and ACE activity in men not taking ACEI and in all studied patients. We have also found a negative correlation between ACE activity and Pmax ( Figure 1) as well as with Ʋopt ( Figure 2) in group of patients not taking ACEI and in the whole group of men ( Table 2). Similar relationships were observed in group of men not taking any ASBMs – both Pmax (borderline significance) and Ʋopt were negatively correlated with serum ACE activity ( Table 2).

**Table 2.** Correlations between ACE activity with anthropometric data, muscle function characteristics and functional performance in men taking/not taking ACEI and taking/not taking ASBMs.

Characteristics	ACEI (n=26)	non ACEI (n=53)	ASBMs (n=34)	non ASBMs (n=45)	all (n=79)
Age (years)	Rho=0.16 P=0.4	Rho=0.38 P=0.006	Rho=0.19 P=0.27	Rho=0.38 P=0.01	Rho=0.26 P=0.02
Body mass (kg)	Rho=-0.15 P=0.45	Rho=0.17 P=0.23	Rho=-0.04 P=0.80	Rho=0.12 P=0.42	Rho=0.04 P=0.7
Body mass index (kg·m ^-2^)	Rho=0.01 P=0.95	Rho=0.10 P=0.46	Rho=0.10 P=0.56	Rho=0.04 P=0.81	Rho=0.02 P=0.84
Medications (number)	Rho=0.24 P=0.24	Rho=0.09 P=0.50	Rho=0.17 P=0.33	Rho=0.001 P=0.99	Rho=0.03 P=0.77
Fat percentage of body mass (%)	Rho=-0.21 P=0.31	Rho=0.19 P=0.19	Rho=-0.07 P=0.70	Rho=0.14 P=0.38	Rho=0.04 P=0.75
Left handgrip strength (kg)	Rho=-0.40 P=0.087	Rho=-0.22 P=0.12	Rho=-0.29 P=0.10	Rho=-0.21 P=0.18	Rho=-0.22 P=0.06
Right handgrip strength (kg)	Rho=-0.18 P=0.38	Rho=-0.14 P=0.32	Rho=-0.08 P=0.64	Rho=-0.15 P=0.32	Rho=-0.12 P=0.29
Pmax (W·kg ^-1^)	Rho=-0.19 P=0.34	**Rho=-0.29** **P=0.04**	Rho=-0.14 P=0.42	Rho=-0.29 P=0.06	Rho=-0.21 P=0.06
Ʋopt (rep/min)	Rho=-0.15 P=0.45	**Rho=-0.31** **p=0.03**	Rho=-0.16 P=0.35	**Rho=-0.31** **P=0.04**	**Rho=-0.22** **P=0.05**
ADL	Rho=-0.22 P=0.27	Rho=-0.04 P=0.76	Rho=-0.18 P=0.31	Rho=-0.002 P=0.99	Rho=-0.06 P=0.58
IADL	Rho=0.06 P=0.77	Rho=-0.07 P=0.60	Rho=0.18 P=0.29	Rho=-0.09 P=0.54	Rho=0.09 P=0.42
TUG (s)	Rho=-0.04 P=0.85	Rho=-0.12 P=0.39	Rho=-0.13 P=0.46	Rho=-0.11 P=0.47	Rho=-0.13 P=0.27

Abbreviations: Pmax, maximum power; Ʋopt, optimal shortening velocity of the knee extensor muscles; ACE, angiotensin-converting enzyme; ACEI, Angiotensin Converting Enzyme Inhibitors; ASBMs, angiotensin system blocking medications; TUG, Timed Up and Go test; ACEI - men taking ACEI; non ACEI – men not taking ACEI; ASBMs - men taking ASBMs; non ASBMs - men not taking ASBMs

However, when ACE activity and age were entered into the multiple regression analyses, age was the only selected variable to determine both Pmax and Ʋopt.

## Discussion

In this study we have shown that muscle power and muscle contraction velocity were negatively correlated with ACE activity in patients not taking ACEI and in patients not taking any ASBMs. However, taking ACEI was not consistently associated with handgrip strength, muscle power, muscle contraction velocity, and functional performance in older men of the same age and with comparable anthropometric parameters.

In 2002, Onder *et al.* ^7^ published an article suggesting that treatment with ACEI may be associated with slower decline in muscle strength and function in older women with hypertension. During the three-year observation the reduction in the muscle strength of the knee extensors and in walking speed was significantly lower in women who continuously used ACEI than in those who used other or did not used any hypertensives. Buford *et al.* also reported greater exercise-derived improvements in physical function for older ACEI users ^24^. In EELO Project, taking medicines modulating the effect of angiotensin II (both ACEI and angiotensin receptors blockers) was connected with better functional capacity and muscle strength in people over 60 year of age ^25^. In the present study, despite the fact that patients receiving any ASBMs were more than two years older than those who did not take ASBMs, they did not differ in terms of muscle power and Ʋopt. This is consistent with our last study in older women ^14^. Women taking ACEI were almost two years older, had lower health status than women not taking ACEI, but these two groups did not differ in terms of handgrip strength, Pmax, Ʋopt and functional performance. These results may suggest effect of RAS system on muscle performance.

However, there are some studies which do not confirm clearly the protective effect of ACEI on muscle and functional performance and on improvement of the response to exercises training. Sumukadas *et al.* ^26^ did not find effects of ACEI therapy on functional status as well as on improvement in handgrip and quadriceps muscle strength in older people undergoing 20-week progressive exercise training in comparison with placebo. In nine-month follow-ups taking ACEI was connected with decreasing of ACE activity but there was no association between the changes in ACE activity and changes in muscle strength or functional capacity in older community dwelling subjects ^27^. Similarly, in the TRAIN study, the authors did not observe significant modifications in physical performance and handgrip strength after six-months of fosinopril use in older persons with high cardiovascular risk profile ^28^. In 4.4 years, a follow-up study ^29^ taking any from cardiovascular drugs (ACEI, statins or thiazides) was not associated with differences in handgrip strength decline in healthy older people.

Therefore, the results regarding the relationship between ACEI and maintaining/improving muscle mass and strength in older age are still inconsistent. Many additional factors can potentially influence this relationship, like concomitant diseases, the type and dosage of the drugs, genetic predisposition, lifestyle, etc. The key point may be the observation time. The longest period of observations mentioned above was 4.4 years ^29^, but some of them were shorter - even six months ^28^. Decreasing muscle mass and function is not a rapid process. It has been reported that the strength loss in older men is 2–3.4% per year and is modulated by some other factors ^30^. Moreover, in one observation 15% of subjects older than 60 did not at all decline handgrip strength during the average nine-year follow-up ^31^. Therefore, taking ACEI may be too weak a factor to notice its impact on maintaining muscle mass and function in a relatively short period of time and a longer follow-up is needed to confirm the importance of ACEI in this area.

This seems also likely in the context of our research. The main effect of ACEI use is the decrease of ACE activity ^8^. Our results show that men with higher serum ACE activity are characterized by lower Pmax as well as lower Ʋopt. These correlations apply only to groups of patients not taking ACEI, not taking any ASBMs and all studied men, but not men taking ACEI or ASBMs. In men taking ACEI or other ASBMs in whom pharmacotherapy modulated ACE activity, this relationship is not clear. We also did not observe a similar relationship in relation to the handgrip strength. Interestingly, even if the relationships between ACE activity and muscle function (strength, power, contraction velocity) did not reach statistical significance, the direction of the association has always been the same - lower ACE activity was associated with better muscle function. This observation applies to all indicators of muscle function and to all analyzed patients’ subgroups in both our latest ^14^ and current study. Statistically significant dependence in our last work in older women was demonstrated only for ACE activity and Ʋopt ^14^. In the current study, in addition to Ʋopt also Pmax is negatively correlated with ACE activity, but handgrip strength is not. Ʋopt may be a more sensitive indicator of muscle function than strength and Pmax (force and velocity indicator), decreases with age faster than strength ^13^. Our male group is older than previous group of women. Therefore, higher ACE activity had a chance to have a longer impact on the muscles. In both male groups (taking and not taking ACEI) higher ACE activity was also seen as compared to women.

This data seems also consistent with studies based on genetic tests. Serum ACE concentration is genetically determined (an insertion/deletion – I/D polymorphism in the ACE gene) ^9^, so depending on the genetic predisposition, reduced or increased ACE activity has been acting on the studied men for their entire life. ACE genotype and activity may be not related to muscle function tests in young subjects ^32^. In contrast, among older people deletion polymorphism of ACE gene (DD), connected with higher level of angiotensin II, was associated with low muscle mass ^33^. Similarly, advanced cancer patients with DD ACE gene polymorphism were characterized by higher serum ACE activity but lower hand grip strength compared with ACE insertion group ^34, 35^.

There are several mechanisms that can explain the effects of ACE activity on Ʋopt and Pmax. ACE is responsible for the conversion of angiotensin I (ang-I) to angiotensin II (ang-II). Ang-II has vasoconstrictor effect, may decrease muscle blood flow and impair the delivery of insulin and glucose to skeletal muscle. The hyper-activity of RAS system may be therefore with an increased ACE activity, and thus with the hyper-activity of the RAS system. This hyper-activity may be associated with tissue insulin resistance ^36^. In addition it may also stimulate mitochondrial dysfunction, oxidative stress, and promote inflammatory processes ^36^ what may be connected with muscle atrophy. Ang-II also induces protein degradation through reactive oxygen species accumulation ^36^. Thus, lower ACE activity may limit muscle atrophy and the associated decline in muscle function (strength, power, contraction velocity). Lower ACE activity also have a neuroprotective effect ^37, 38^ what is crucial because neural mechanisms are important factors in the development of sarcopenia ^39^.

Conditions such as heart failure, renal failure, chronic obstructive pulmonary disease, and cancer are associated with increased activation of the RAS pathway. These conditions can contribute to muscle atrophy, and thus also the decline in muscle function ^36^.

Since ACE activity in our study was positively correlated with age, we decided to conduct multiple regression analyses for determinants of both Pmax and Ʋopt including ACE activity and age as independent variables. In these analyses age was the only selected variable determining Pmax and Ʋopt. The positive relationship between ACE activity and age was surprising, but there were no studies in such advanced age so far. In a few works on this subject, serum ACE activity increased with age in children up to 18 years old ^40^ but did not vary with age in adults ^41, 42^ or even decreased in rats ^43^ and in adult men ^44^.

### Limitation of the study

This study has some limitations. First, the ACEI intake period was not analyzed. We only recorded taking or not taking ACEI and other medications. Therefore, it is worth conducting research taking into account the time of taking medications, because the duration of the effect of drugs on the muscles may be important in this case.

When interpreting the data, it should be taken into account that men taking ACEI took more medications and performed worse in the assessment of functional status in the IADL scale. This may indicate the poorer health status of these men and affect the interpretation of the results related to muscle function (muscle function may be determined by health status not only by ACE level). On the other hand, the relationship between serum ACE levels and muscle function occurred only in the non-ACEI group, i.e. in the group in which the drugs did not modify ACE levels. Finally, due to the size of the group and the limitations mentioned above, this work should be regarded as preliminary results that require further research, given the potential role of ACE in aging.

## Conclusions

Serum ACE activity negatively determines muscle power and muscle contraction velocity but not muscle strength in older men. The issues related to the impact of taking ACEI on the maintenance of muscle function and functional performance in older man require further studies with long-term follow-up, and determining the type and dose of the treatment.

## Data availability

### Underlying data

Zenodo: Quadriceps muscle power and optimal shortening velocity are inversely related to angiotensin converting enzyme activity in older men, http://doi.org/10.5281/zenodo.4493095 ^45^.

Data are available under the terms of the Creative Commons Attribution 4.0 International license (CC-BY 4.0).
