# Supplementary material for: Quadriceps muscle power and optimal shortening velocity are inversely related to angiotensin converting enzyme activity in older men
Source: F1000Res. 2021 Jun 9;10:184. Originally published 2021 Mar 5. [Version 2] doi: 10.12688/f1000research.51208.2 (PMC8287535; doi:10.12688/f1000research.51208.2)
Supplement: Peer review history

# Peer review: Reviewer response for version 2

**DOI:** 10.5256/f1000research.57350.r87203

**Published:** 2021-07-16

**Authors:** Christophe A. Hautier

**Affiliations:** 1 Laboratoire Interuniversitaire de Biologie de la Motricité, Université Claude Bernard Lyon 1, Villeurbanne, France

I think this article has been greatly improved, especially with the addition of the limitations part and with the appropriate details on the methodology.

 I think it can be indexed as is because although the conclusions are still a bit speculative, the subject and the approach deserve to be developed in future studies.

Is the work clearly and accurately presented and does it cite the current literature?

Yes

If applicable, is the statistical analysis and its interpretation appropriate?

Yes

Are all the source data underlying the results available to ensure full reproducibility?

Yes

Is the study design appropriate and is the work technically sound?

Yes

Are the conclusions drawn adequately supported by the results?

No

Are sufficient details of methods and analysis provided to allow replication by others?

No

Reviewer Expertise:

Sport Sciences, physiology, biomechanics

I confirm that I have read this submission and believe that I have an appropriate level of expertise to confirm that it is of an acceptable scientific standard.

# Peer review: Reviewer response for version 1

**DOI:** 10.5256/f1000research.54351.r81773

**Published:** 2021-05-17

**Authors:** Christophe A. Hautier

**Affiliations:** 1 Laboratoire Interuniversitaire de Biologie de la Motricité, Université Claude Bernard Lyon 1, Villeurbanne, France

This article focused about the effects of angiotensin Converting Enzyme Inhibitors (ACEI) and other angiotensin system blocking medications (ASBMs) on the strength and speed capacities of the skeletal muscle in the elderly. The subject is remarkably interesting in the prevention of dependence in the elderly because the means of combating sarcopenia and dynapenia are still poorly understood. Of course, exercise and diet have a significant effect on the muscle mass and strength and functionality of the elderly, on the other hand it seems interesting to continue to look for medicinal methods that can help maintain autonomy.

 Seventy-nine elderly men took part in the study, which is still an interesting sample and suggests that the results can be extrapolated to a larger population. Positive relationship between age and ACE activity was demonstrated and, relatively weak negative correlations were observed between ACE activity and Maximum Power or optimal speed. However, taking Angiotensin Converting Enzyme Inhibitors (ACEI) did not modify muscle performance in this population.

 This article is well written however, the rationale of taking converting Enzyme Inhibitors (ACEI) to maintain muscle mass and strength should be reinforced with some mechanistic hypotheses. Even if the authors previously published an article about the relationship of quadriceps muscle power and optimal shortening velocity with angiotensin-converting enzyme activity in older women, there is only indirect evidence generally associating the functional status of the senior and ACE activity.

 The method of constituting the groups of subjects (ACEI vs non ACEI and ASBMs vs non ASBMs) is not presented and moreover the different categories appear only in the results and not in the method. This seems to be an important limitation of this work, for interpreting differences in strength and/or BMI. I think it can be improved a lot by better explaining the constitution of groups in the method part. The fact that patients taking ACEI took more medications and were characterized by lower level of ACE in blood samples and lower IADL status is a major concern that should be better discussed in the limitations of the study. Nevertheless, the correlations are interesting and provide additional evidence about the significance of ACE in ageing.

 I think it is necessary to do a paragraph on the limitations of the study and better discuss the fact that these results are a bit like preliminary results that require further study given the potential role of ACE in aging. I would have liked the authors to touch very lightly on the mechanistic assumptions that might explain the effects of ACE on optimal speed and maximum power. These hypotheses are they neuromuscular, systemic, cardiovascular or else indirect because of the improvement in the quality of life and spontaneous activity. I do not think the authors can conclude on the ineffectiveness of taking ACEI in maintaining functional performance in older men.

Is the work clearly and accurately presented and does it cite the current literature?

Yes

If applicable, is the statistical analysis and its interpretation appropriate?

Yes

Are all the source data underlying the results available to ensure full reproducibility?

Yes

Is the study design appropriate and is the work technically sound?

Yes

Are the conclusions drawn adequately supported by the results?

No

Are sufficient details of methods and analysis provided to allow replication by others?

No

Reviewer Expertise:

Sport Sciences, physiology, biomechanics

I confirm that I have read this submission and believe that I have an appropriate level of expertise to confirm that it is of an acceptable scientific standard, however I have significant reservations, as outlined above.

# Response

**Published:** 2021-05-28

**Authors:** Joanna Kostka

**Affiliations:** Medical University of Lodz, Poland

Dear Reviewer,

 We would like to thank you for the analysis of this manuscript and your valuable remarks. The manuscript has been corrected according to the comments. All changes are described below:

**1:** *"This article is well written however, the rationale of taking converting Enzyme Inhibitors (ACEI) to maintain muscle mass and strength should be reinforced with some mechanistic hypotheses. Even if the authors previously published an article about the relationship of quadriceps muscle power and optimal shortening velocity with angiotensin-converting enzyme activity in older women, there is only indirect evidence generally associating the functional status of the senior and ACE activity."*

**Answer: **We supplemented the Introduction section with suggested information:

 The authors of the article explain this effect with several mechanisms, such as: direct influence on mechanical and metabolic changes in muscles (shift of the myosin heavy chains of skeletal muscle toward the fatigue-resistant forms; increasing insulin sensitivity, glycogen storage and glucose uptake in skeletal muscle; reduction of kinin breakdown and, consequently, improved circulation and better glucose and amino acid uptake), suppression of inflammatory activation connected with muscle catabolism, beneficial effect on the nutritional status (inhibition of interleukin-6, which, among others, reduces appetite and, consequently, may cause malnutrition).

**2:** *"The method of constituting the groups of subjects (ACEI vs non ACEI and ASBMs vs non ASBMs) is not presented and moreover the different categories appear only in the results and not in the method. This seems to be an important limitation of this work, for interpreting differences in strength and/or BMI. I think it can be improved a lot by better explaining the constitution of groups in the method part."*

**Answer: **Suggested information has been added to the “Methods” section:

 Based on the analysis of medications taken, men were assigned to the following groups: ACEI - if they were taking any medications belonging to the ACEI group (n = 26) and to the non ACEI group - if they were not taking ACEI (n = 53). Similarly, if men were taking any drugs from the angiotensin system blocking medications (ASBMs) group, they were assigned to the ASBMs group (n = 34), if not, then to the non ASBMs group (n = 45).

**3.** *"The fact that patients taking ACEI took more medications and were characterized by lower level of ACE in blood samples and lower IADL status is a major concern that should be better discussed in the limitations of the study."*

**Answer: **This problem is discussed in the section “Limitation of the study” as suggested.

**4.** *"I think it is necessary to do a paragraph on the limitations of the study and better discuss the fact that these results are a bit like preliminary results that require further study given the potential role of ACE in aging."*

**Answer: **A "Limitation of the study" section was added to the discussion.

 Limitation of the study

 This study has some limitations. First, the ACEI intake period was not analyzed. We only recorded taking or not taking ACEI and other medications. Therefore, it is worth conducting research taking into account the time of taking medications, because the duration of the effect of drugs on the muscles may be important in this case.

 When interpreting the data, it should be taken into account that men taking ACEI took more medications and performed worse in the assessment of functional status in the IADL scale. This may indicate the poorer health status of these men and affect the interpretation of the results related to muscle function (muscle function may be determined by health status not only by ACE level). On the other hand, the relationship between serum ACE levels and muscle function occurred only in the non-ACEI group, i.e. in the group in which the drugs did not modify ACE levels. Finally, due to the size of the group and the limitations mentioned above, this work should be regarded as preliminary results that require further research, given the potential role of ACEs in aging.

**5.** *"I would have liked the authors to touch very lightly on the mechanistic assumptions that might explain the effects of ACE on optimal speed and maximum power. These hypotheses are they neuromuscular, systemic, cardiovascular or else indirect because of the improvement in the quality of life and spontaneous activity."*

**Answer: **We slightly modified and expanded part of the discussion on this topic:

 There are several mechanisms that can explain the effects of ACE activity on Ʋopt and Pmax potentially affecting muscle function that are associated with an increased ACE activity, and thus with the hyper-activity of the RAS system. ACE is responsible for the conversion of angiotensin I (ang-I) to angiotensin II (ang-II). Ang-II has vasoconstrictor effect, may decrease muscle blood flow and impair the delivery of insulin and glucose to skeletal muscle. The hyper-activity of RAS system may be therefore associated with tissue insulin resistance ^36^, muscle atrophy, and fibrosis. RAS In addition it may also stimulate mitochondrial dysfunction, oxidative stress, and promote inflammatory processes ^36^ what may be connected with muscle atrophy. Ang-II also induces protein degradation through reactive oxygen species accumulation ^36^. Thus, lower ACE activity may limit muscle atrophy and the associated decline in muscle function (strength, power, contraction velocity). Lower ACE activity may also have a neuroprotective effect ^37, 38^ what is crucial because neural mechanisms are important factors in the development of sarcopenia ^39^ .

 Conditions such as heart failure, renal failure, chronic obstructive pulmonary disease, and cancer are associated with increased activation of the RAS pathway. These conditions can contribute to muscle atrophy, and thus also the decline in muscle function ^36^. 

**6.** *"I do not think the authors can conclude on the ineffectiveness of taking ACEI in maintaining functional performance in older men."*

**Answer: **We modified the conclusions (also in the abstract):

 The issues related to the impact of taking ACEI on the maintenance of muscle function and functional performance in older man require further studies with long-term follow-up, and determining the type and dose of the treatment.

 Best regards

 Joanna Kostka

# Peer review: Reviewer response for version 1

**DOI:** 10.5256/f1000research.54351.r81534

**Published:** 2021-03-29

**Authors:** Ida Wiszomirska

**Affiliations:** 1 Faculty of Rehabilitation, Józef Piłsudski University of Physical Education, Warsaw, Poland

Review of the article “Quadriceps muscle power and optimal shortening velocity are inversely related to angiotensin-converting enzyme activity in older men.”

 The topic undertaken in this article is important. Aging is associated with a gradual loss of muscle function and, consequently, deterioration of functional efficiency. Identification of factors that could slow down this process may contribute to the improvement of the functioning of the elderly.

**Introduction**

 The introductory part contains a factual and correct justification of the topic undertaken in the study, taking into account correctly selected items of the literature. The objectives of the work formulated correctly.

 One sentence requires an explanation:

- " *Taking ACEI as well as the ACE genotype with insertion (II) of allele in gene, is associated with lower serum ACE activity and with predispositions for practicing some sports*." – there is no evidence, that taking ACEI is associated with predispositions for practicing some sports (in fact, there are some reports about the ACE genotype) - this sentence suggests such a relationship. Please formulate this sentence in a different way.

**Methodology**

 Methodology is generally well described, but it is worth supplementing some information:

- Please add "for each hand separately" to the sentence " *A better record was used for analysis"* (last sentence of the section " *Muscle strength"*).
- Despite the reference to the literature, in this part it is worth describing the measurement of Pmax and Ʋopt in a little more detail.

Results are shown as a text, in tables and in 2 figures. This section is presented clearly.

 In the discussion, the results of own research were compared to the current literature. In this section, it is worth discussing the limitations of the study. Correctly formulated conclusions.

Is the work clearly and accurately presented and does it cite the current literature?

Yes

If applicable, is the statistical analysis and its interpretation appropriate?

Yes

Are all the source data underlying the results available to ensure full reproducibility?

Yes

Is the study design appropriate and is the work technically sound?

Yes

Are the conclusions drawn adequately supported by the results?

Yes

Are sufficient details of methods and analysis provided to allow replication by others?

Yes

Reviewer Expertise:

Physiotherapy, Physical Rehabilitation, Muscle Function

I confirm that I have read this submission and believe that I have an appropriate level of expertise to confirm that it is of an acceptable scientific standard.

# Response

**Published:** 2021-05-28

**Authors:** Joanna Kostka

**Affiliations:** Medical University of Lodz, Poland

Dear Reviewer,

 We would like to thank you for the analysis of this manuscript and your valuable remarks. The manuscript has been corrected according to the comments. All changes are described  below.

**1.** *One sentence requires an explanation:*

*"Taking ACEI as well as the ACE genotype with insertion (II) of allele in gene, is associated with lower serum ACE activity and with predispositions for practicing some sports." – there is no evidence, that taking ACEI is associated with predispositions for practicing some sports (in fact, there are some reports about the ACE genotype) - this sentence suggests such a relationship. Please formulate this sentence in a different way.*

**Answer: **This sentence was modified to:

 “Taking ACEI as well as the ACE genotype with insertion (II) of allele in gene, is associated with lower serum ACE activity ^8,9^. There are suggestions that the ACE genotype type predisposes one to be successful in certain sports ^10^.”

**2**. *Methodology*

*Please add "for each hand separately" to the sentence "A better record was used for analysis" (last sentence of the section "Muscle strength").*

**Answer: **We have added this sentence in line with the Reviewer's comment.

**3**. *Despite the reference to the literature, in this part it is worth describing the measurement of Pmax and Ʋopt in a little more detail.*

**Answer: **The description of the Pmax and Ʋopt measurement methodology has been supplemented:

 “Muscle power and optimal shortening velocity evaluations were performed with especially prepared ergometer (Monark type 818E Stockholm, Sweden), during two 8-second attempts to pedal at the maximum possible speed with friction loads of 0.25 N·kg ^−1^ and 0.35 N·kg ^−1^ of body mass. Instantaneous pedaling velocity (Ʋ), force (F) and power output (P) were calculated each 5ms and then averaged over each downstroke period. The highest value of P (maximal short-term power – Pmax) and optimal shortening velocity (Ʋopt - velocity at which the power reaches a maximum value) were calculated from a 3rd order polynomial function. The methodology …”

**4.** *Discussion*

*In this section, it is worth discussing the limitations of the study.*

**Answer: **A "limitation of the study" section was added to the discussion.

 Best regards

 Joanna Kostka
